# The impact of a team-based intervention on the lifestyle risk factor management practices of community nurses: outcomes of the community nursing SNAP trial

**DOI:** 10.1186/1472-6963-13-54

**Published:** 2013-02-09

**Authors:** Bibiana C Chan, Upali W Jayasinghe, Bettina Christl, Rachel A Laws, Neil Orr, Anna Williams, Kate Partington, Mark F Harris

**Affiliations:** 1 Centre for Primary Health Care and Equity, University of New South Wales, Sydney, Australia; 2 Centre for Physical Activity and Nutrition Research, Deakin University, Melbourne, Australia; 3 Centre for Epidemiology and Evidence, New South Wales Ministry of Health, Sydney, Australia; 4 South West Sydney Local Health District, Sydney, Australia

**Keywords:** Primary health care, Community nursing, Lifestyle risk factor management, Barriers

## Abstract

**Background:**

Lifestyle risk factors like smoking, nutrition, alcohol consumption, and physical inactivity (SNAP) are the main behavioural risk factors for chronic disease. Primary health care is an appropriate setting to address these risk factors in individuals. Generalist community health nurses (GCHNs) are uniquely placed to provide lifestyle interventions as they see clients in their homes over a period of time. The aim of the paper is to examine the impact of a service-level intervention on the risk factor management practices of GCHNs.

**Methods:**

The trial used a quasi-experimental design involving four generalist community nursing services in NSW, Australia. The services were randomly allocated to either an intervention group or control group. Nurses in the intervention group were provided with training and support in the provision of brief lifestyle assessments and interventions. The control group provided usual care. A sample of 129 GCHNs completed surveys at baseline, 6 and 12 months to examine changes in their practices and levels of confidence related to the management of SNAP risk factors. Six semi-structured interviews and four focus groups were conducted among the intervention group to explore the feasibility of incorporating the intervention into everyday practice.

**Results:**

Nurses in the intervention group became more confident in assessment and intervention over the three time points compared to their control group peers. Nurses in the intervention group reported assessing physical activity, weight and nutrition more frequently, as well as providing more brief interventions for physical activity, weight management and smoking cessation. There was little change in referral rates except for an improvement in weight management related referrals. Nurses’ perception of the importance of ‘*client and system-related*’ barriers to risk factor management diminished over time.

**Conclusions:**

This study shows that the intervention was associated with positive changes in self-reported lifestyle risk factor management practices of GCHNs. Barriers to referral remained. The service model needs to be adapted to sustain these changes and enhance referral.

**Trial registration:**

ACTRN12609001081202

## Background

Lifestyle risk factors such as smoking, poor nutrition, at-risk alcohol consumption and physical inactivity (SNAP) have been identified as the main preventable risk factors for chronic diseases worldwide [1]. Reducing the prevalence of these risk factors in the population is important given that chronic disease accounts for more than 60% of the overall global burden of disease [2]. It is well recognised that to reduce the prevalence of behavioural risk factors, a wide range of interventions are required, be they related to policy, the environment or health service intervention.

Primary health care (PHC) has been identified as a suitable setting to address behavioural risk factors. This relates to its accessibility and its capacity for repeated contacts with clients, which provides an opportunity to assess lifestyle risk factors, monitor progress and refer to other health professionals [3]. The evidence suggests that PHC can be appropriate for the delivery of brief lifestyle interventions as it has been shown to improve the rate of smoking cessation [4] and reduce ‘at-risk alcohol’ consumption [5]. Moderate to high intensity interventions provided through PHC also show promise for improving weight, diet and physical activity for those at high risk of developing or progressing in chronic disease [6-8].

**Figure 1:**
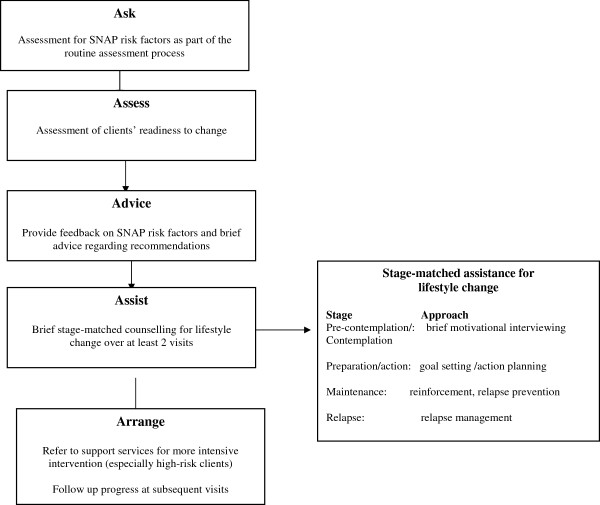
5As model of brief lifestyle intervention using the transtheoretical model of behaviour change.

Despite the beneficial effects of risk factor management, brief lifestyle interventions are rarely implemented in PHC. It appears that assessment for lifestyle risk factors does not occur routinely and only a minority of clients receive any intervention in PHC relating to the prevention or management of chronic disease [9-12]. Furthermore, lifestyle interventions tend to be limited to asking and advising on the risks of the behaviour rather than providing assistance, referral and follow-up (the essential components of a behavioural risk modification intervention) [13-15] (Figure 1).

Much of the focus of research on lifestyle risk factor management in Australian PHC has been in general practice, with little attention being paid to the other PHC settings. In 2011 there were 13,939 nurses employed in state funded community health services compared to a total of 12 576 health professionals employed in general practice [16]. Nurses working for community health services are employed in different roles across the sector. GCHNs provide care for patients recently discharged from hospital, the aged and those with chronic diseases. Although their role has been poorly researched they are in a strong position to offer lifestyle risk factor management as they [17-19]:

1. Usually see clients in their own home, allowing them to develop a broader understanding of the client’s family and home environment that may benefit from lifestyle change;

2. usually have ongoing contact with patients;

3. may reach disadvantaged populations who would otherwise have limited contact with GPs; and

4. have a strong professional ethic which privileges holistic care over a strict medical model.

Our previous work has demonstrated that there is a significant need for brief lifestyle interventions among the clients of GCHNs, as they carry a large burden of chronic disease and associated risk factors and these clients may also be open to changing their risk-related lifestyle practices [20]. In our previous research, we argued that to increase the implementation of brief interventions, an intervention needed to be developed to build positive attitudes, skills and self-efficacy with regard to brief interventions as well as provide organisational level support with decision support tools, improved access to referral services, and resource materials for nurses and clients [21]. An intervention designed around these principles was tested in the Community Nurse SNAP Trial. The aim of this paper is to examine the effectiveness of a team-based intervention on changing the self-reported lifestyle risk factor management practices of GCHNs.

## Methods

This paper is one component of reporting from the Community Nursing SNAP trial, the details of which have been described previously [22].

### Recruitment of services

Four community health services in NSW were recruited to take part in the study. The services were located in regional/semi-rural community health sites with residents from predominately lower socio-economic status (SES) backgrounds. Services were randomly allocated to an ‘early intervention’ or ‘late intervention’ group. Randomisations were performed by a person independent of the research team. An additional team within a site with a low client response rate was added to the early intervention group while the study was in progress to boost client recruitment. When the early and late intervention sites were compared they were found to be of a similar size and to be located in areas with comparable levels of socio-economic disadvantage. All community nurses working in the sites were considered to be part of the study.

### Team-based SNAP intervention

The intervention aimed to increase the capacity of the GCHNs to undertake brief SNAP assessment and intervention and was based on a previously developed model designed for the delivery of brief lifestyle interventions in community health settings. This model is based on the premise that lifestyle risk factor management practices in the community nursing setting are shaped by:

1. clinician commitment, in particular beliefs about role congruence (how nurses’ expectations of their role match with the demand placed on them within the health system) and client receptiveness; and

2. clinician capacity, i.e. self-efficacy, role support and the extent to which lifestyle risk factor management activities fit within clinicians’ current practice.

The intervention was comprised of:

a) a one-day group training program for nurses (delivered by the research team in conjunction with local providers). The training focused on building nurse knowledge, skills and positive attitudes relevant to the constructs of the 5As model [3]. There was a particular focus on developing nurses’ skills in motivational interviewing and goal setting. Emphasis was placed on experiential learning through the use of role-plays with simulated clients (actors), group discussions and activities;

b) the integration of standard assessment tools and prompts for SNAP and weight risk factors into the routine assessment process undertaken by all GCHNs on initial visits with clients;

c) the development of a local service referral directory for each GCHN team, to promote referral of appropriate clients for ongoing specialist management or more intensive and ongoing lifestyle intervention and follow-up (such as Quitline); and

d) the provision of resources to support the implementation of the intervention (e.g. written guides for nurses, written action plans for each SNAP risk factor, tape measures and pedometers to lend out to clients to encourage self-monitoring).

A nurse from each of the early intervention sites was seconded to work with the research team to support implementation of the study at the local level. ‘Late intervention services’ (control) continued to deliver usual care, thus acting as the comparison group. Late intervention services were offered the intervention after all outcome data had been collected.

### A. Quantitative data collection and analysis

#### Data collection

Self-reported lifestyle risk factor management confidence, attitudes and practices of GCHNs were measured at baseline, six and twelve months. Data was collected using an on-line survey (Additional file 1) adapted from the Preventive Medicine Attitudes and Activities Questionnaire (PMAAQ) [23]. Before the survey was disseminated, it was pilot tested with a group of community nurses who were not part of the trial The survey examined how often the nurses assessed lifestyle risk factors and provided brief intervention and/or referral as part of their routine practice, using a Likert scale of 1 – 7 from ‘*never*’ to ‘*always*’ (e.g. *How often on average do you do the following when dealing with a client who smokes?*). The survey also assessed nurse confidence and attitudes to the management of lifestyle risk factors using a Likert scale of 1 – 5 (‘*Not at all confident’* to ‘*very confident*’). Nurses were given two weeks to complete the survey and non-responders were given a reminder two weeks after it was initially distributed. After a further two weeks, paper copies were sent to non-responders on the assumption that they were unable to access a computer to complete the on-line survey.

#### Data analysis

Factor analysis was conducted on survey items to reduce the number of pre-and-post item comparisons. This was undertaken using principal axis factor analysis and a number of factors were identified by the *scree* test [24], which became the basis of the pre-post comparisons. The results of the factor analysis indicated that intervention items could be integrated into one variable for each risk factor. Further, ‘perceived barriers’ items could be combined into three components. These were:

1. ‘client & support related barriers’ (e.g. *lack of client interest in lifestyle changes*);

2. ‘clinician-related barriers’ (e.g. *lack of relevance to my role*); and

3. ‘service-related barriers’ (e.g. *pressures of providing post-acute care*).

A score was calculated for each risk factor by totalling the scores for each assessment or management item and dividing by the number of items. Outcomes using repeated measures analysis of variance (ANOVA) were then examined in relation to assessment and intervention scores and the three barrier components. Each outcome was tested for change over time (baseline, six, and twelve months) and for differences in the time course between the two groups by assessing the main effect of time and the group-by-time interaction with repeated measure ANOVA. Repeated measure analyses were not used for the reported frequency of referrals because these were all single item scores and repeated measure was more suited for comparison of average scores derived from multiple items. We computed Cohen’s d effect sizes. Cohen defined an effect size of 0.20 as small, one of 0.50 as moderate, and one of 0.80 or greater as large [25]. The effect size could be used as a specific benchmark or standard for interpreting difference in changes between two groups. Data were analysed using SPSS 18 [26].

Changes in nurses’ confidence in conducting SNAP risk factor assessment and intervention were analysed using paired t-tests comparing mean scores between baseline and six months and baseline and 12 months.

### B. Qualitative data collection and analysis

#### Data collection

All community nurses who participated in the intervention (n=71 early intervention sites only) were invited to take part in a focus group. The focus groups aimed to (1) explore nurses’ experiences of integrating lifestyle risk factor management into their current work; (2) ascertain the nurses’ views on how useful the intervention was; and (3) learn about any problems the nurses had encountered in delivering the intervention, in particular problems that might reduce the sustainability of providing the intervention as part of routine care. A project officer at each site allocated interested nurses in their team to attend a focus group best suited to their work schedule. The nursing unit managers also encouraged nurses to support the study at their team meetings and through follow-up emails. One researcher facilitated each group and a second researcher observed and took notes of group interactions. The nurse unit managers (n=4) and project officers (n=2) at early intervention sites were also invited to take part in a face-to-face semi-structured interview. Focus group participants and interviewees signed consent forms prior to the interviews. All focus groups and interviews were audio-taped with the participants’ consent.

#### Data analysis

Qualitative data (focus group narratives and individual interviews) were transcribed verbatim and coded using NVivo 8 [27]. As the lifestyle interventions were designed based on the 5As model, coding focused on the acceptability and perceived usefulness of the SNAP intervention across the 5A components. One researcher conducted the initial coding. Subsequently 15% of the data was then cross-coded by a second researcher, with a final comparison of the two coding frames conducted. Disparities in coding between the researchers were discussed until consensus was reached. A thematic analysis was then performed.

### Ethics

The project was approved by the Human Research Ethics Committees at UNSW and HREC Committees within all of the former Area Health Services in which the study was conducted.

## Results

### On-line survey

The response rate for the baseline survey was 72.5% (129/178) and retention rates at six months and twelve months were 62.8% (81/129) and 50.4% (65/129) respectively. This gave an overall response rate of 41.9% (54/129) across all time points. The majority of participants were registered nurses, female, aged 35–54 years and working full-time (Table 1). Approximately half of the participants (54.7%) had been working in their current position for over 5 years (mean: 8.2 years; SD: 7.3 years), although the range was six months to twenty five years. The nurses had worked as community nurses for an average of thirteen years (SD: 10.9 years; range 0.5–39 years).

**Table 1:** Characteristics of CN SNAP Trial nurses completing baseline survey

**Characteristics**	**N = 54**	
**Gender**	N	%
Female	51	94.4%
Male	3	5.6%
Age (years)*		
18-34	10	18.8%
35-54	28	52.8%
≥ 55	15	28.3%
Employment status*		
Full-time	35	64.8%
Part-time	18	33.3%
Qualification*		
Registered Nurse	50	94.3%
Enrolled Nurse	3	5.7%
Length of Service(working as a community nurse)*		
≤ 10 year	26	49.1%
11 – 20 year	14	26.4%
>20 year	13	24.5%
Length with the current team*		
≤ 5 years	24	45.3%
6-10 years	12	22.6%
>10 years	17	32.1%

* data missing from one participant.

#### SNAP assessment

Repeated measure analyses of variance comparing the early intervention (hereafter ‘***intervention***’) and late intervention (hereafter ‘***control***’) groups on all five assessment scores at baseline, six and twelve months (see Table 2) showed no significant change in the frequencies in assessing smoking. There were positive associations between the intervention group and increased frequencies in assessment for nutrition, alcohol and weight.

**Table 2:** Nurses’ reported assessment, management and referral at baseline, 6 months and 12 months

	**Group mean (95% confidence interval)**		***p***^**1**^			
***Risk factor***	***Intervention***	***Control***	***Group***	***Time***	***Group × time interaction***	**Effect size (95% CI)**
**Assessment**						
**Smoking**	(*n* = 31)	(*n* =22 )	0.670	0.794	0.245	
Baseline	5.94 (5.32-6.55)	6.18 (5.61-6.76)				−0.16 (−0.70–0.39)
6-month	6.07 (5.42-6.71)	5.81 (5.11–6.52)				0.15 (−0.40–0.69)
12- month	6.16 (5.59-6.71)	5.68 (4.93–6.44)				0.30 (−0.26–0.84)
**Nutrition**	(*n* = 32)	(*n* = 22 )	0.080	0.242	**<0.001**	
Baseline	5.22 (4.54–5.90)	5.27 (4.64–5.90)				−0.03 (−0.57–0.51)
6-month	5.63 (4.94–6.31)	5.18 (4.50–5.86)				0.26 (−0.29–0.80)
12- month	5.91 (5.41–6.40)	4.18 (3.40–4.96)				1.12 (0.52–1.69)
**Alcohol**	(*n* =30)	(*n* = 20)	**0.042**	0.891	0.703	
Baseline	5.73 (5.05–6.41)	5.00 (4.10–5.90)				0.39 (−0.18–0.96)
6-month	6.00 (5.35–6.65)	4.90 (3.97–5.84)				0.60 (0.01–1.16)
12- month	6.00 (5.36–6.64)	4.95 (3.9 –5.93)				0.56 (−0.02–1.13)
**Physical activity**	(*n* = 32)	(*n* = 22)	0.563	**<0.001**	**<0.001**	
Baseline	3.75 (3.04–4.47)	4.86 (4.14–5.59)				−0.60 (−1.15–-0.04)
6-month	5.56 (4.89–6.24)	4.96 (4.26–5.65)				0.34 (−0.21–0.89)
12- month	5.72 (5.16–6.28)	4.55 (3.79–5.30)				0.72 (0.15–1.27)
**Weight**	(*n* = 32)	(*n* = 21)	0.399	0.348	**0.041**	
Baseline	4.09 (3.29–4.90)	4.48 (3.65–5.30)				−0.19 (−0.74–0.37)
6-month	4.88 (4.09–5.66)	4.57 (3.80–5.34)				0.16 (−0.40–0.70)
12- month	5.06 (4.42–5.70)	3.91 (3.12–4.69)				0.65 (0.08–1.21)
**Intervention**						
**Smoking**	(*n* = 31)	(*n* = 18)	0.497	**0.005**	**<0.001**	
Baseline	3.07 (2.58–3.59)	3.68 (3.08–4.28)				−0.45 (−1.03–0.15)
6-month	4.31 (3.67–4.96)	3.53 (2.85–4.20)				0.48 (−0.12–1.06)
12- month	4.16(3.60–4.71)	3.57 (2.98–4.16)				0.42 (−0.17–1.00)
**Nutrition**	(*n* = 30)	(*n* = 22)	0.934	0.072	0.059	
Baseline	3.96 (3.419–4.50)	4.44 (3.88–5.00)				−0.35 (−0.90–0.21)
6-month	4.67 (4.038–5.32)	4.53 (3.957–5.11)				0.09 (−0.46–0.64)
12- month	4.78 (4.25–5.32)	4.36 (3.75–4.97)				0.30 (−0.26–0.85)
**Alcohol**	(*n* = 30)	(*n* = 21)	0.672	0.115	0.144	
Baseline	3.33 (2.75–3.92)	3.69 (2.99–4.38)				−0.23 (−0.79–0.33)
6-month	4.08 (3.40–4.75)	3.70 (3.09–4.32)				0.23 (−0.33–0.79)
12- month	4.11 (3.52–4.69)	3.64 (2.96–4.32)				0.31 (−0.26–0.86)
**Physical activity**	(*n* = 30)	(*n* = 21)	0.751	**0.007**	**0.007**	
Baseline	3.29 (2.88–3.71)	4.20 (3.57–4.84)				−0.74 (−1.30–-0.15)
6-month	4.53 (3.96–5.11)	4.01 (3.42–4.59)				0.36 (−0.21–0.92)
12- month	4.36 (3.84–4.88)	4.29 (3.57–5.017)				0.05 (−0.51–0.60)
**Weight**	(*n* = 30)	(*n* = 21)	0.932	**0.006**	**0.025**	
Baseline	3.25 (2.75–3.75)	4.11 (3.47–4.75)				−0.63 (−1.19–-0.05)
6-month	4.52 (3.92–5.12)	3.92 (3.41–4.43)				0.42 (−0.15–0.98)
12- month	4.31 (3.85–4.77)	3.95 (3.28–4.63)				0.27 (−0.30–0.82)
**Referrals**			EI Paired t-test* Mean difference (95% CI)		LI Paired t-test* Mean difference (95% CI)	
**Nutrition**	(*n* = 32)	(*n* = 22)	**ΔT**_**0**_ **T**_**1**_**: ΔT**_**1**_ **T**_**2**_		**ΔT**_**0**_ **T**_**1**_**: ΔT**_**1**_ **T**_**2**_	
Baseline	3.84 (3.16–4.52)	3.73 (2.94–4.52)				0.06 (−0.49–0.60)
6-month	4.37 (3.57–5.16)	4.25 (3.42–5.08)	0.52 (−0.23 -1.26)		0.35 (−1.10- 0.90)	0.09 (−0.45–0.63)
12- month	4.47 (3.73–5.20)	4.27 (3.46–5.08)	0.10 (−0.64 - 0.84)		0.10 (−0.90-1.10)	0.10 (−0.45–0.64)
**Alcohol**	(*n* = 32)	(*n* = 22)				
Baseline	3.41 (2.70–4.11)	3.41 (2.65–4.17)				0.0 (−0.54–0.54)
6-month	2.94 (2.25–3.63)	3.76 (2.82–4.70)	−0.47 (−1.37 - 0.43)		0.24 (−0.58-1.05)	−0.39 (−0.93–0.16)
12- month	3.03 (2.31–3.76)	3.29 (2.46–4.11)	0.07 (−0.84 - 0.97)		−0.25 (−1.16-0.66)	−0.13 (−0.67–0.42)
**PA**	(*n* = 32)	(*n* = 22)				
Baseline	2.72 (2.15–3.29)	3.95 (3.14–4.77)				−0.69 (−1.24–-0.12)
6-month	3.19 (2.56–3.81)	3.82 (2.94–4.70)	0.47 (−0.09 - 1.03)		- 0.14 (−0.83-0.55)	−0.33 (−0.87–0.22)
12- month	3.19 (2.53–3.86)	3.76 (2.89–4.64)	−0.07 (−0.62 - 0.49)		−0.19 (−1.55-1.17)	−0.29 (−0.83–0.26)
**Quitline**	(*n* = 32)	(*n* = 22)				
Baseline	3.19 (2.43–3.96)	3.77 (2.91–4.64)				−0.27 (−0.81–0.28)
6-month	4.13 (3.34–4.91)	3.76 (2.84–4.68)	1.03 (−0.01 - 2.07)		0.05 (−0.64-0.73)	0.17 (−0.38–0.71)
12- month	4.35 (3.64–5.07)	3.57 (2.79–4.35)	0.13 (−0.65 - 0.90)		−0.20 (−0.90-0.49)	0.39 (−0.16–0.93)
**Weight management**	(*n* = 32)	(*n* = 22)				
**PA referral**						
Baseline	2.16 (1.60–2.71)	2.86 (2.16–3.57)				−0.43 (−0.97–0.13)
6-month	3.44 (2.74-4.13)	2.77 (2.01–3.53)	***1.28(0.56 -2.00)***		−0.09 (−0.75-0.56)	0.35 (−0.21–0.89)
12- month	3.06 (2.47–3.66)	2.81 (2.03–3.59)	−0.45 (−1.11- 0.21)		0.05 (−0.79-0.69	0.14 (−0.41–0.68)
**Nutrition referral**						
Baseline	3.75 (2.96-4.54)	4.09 (3.26–4.92)				−0.16 (−0.70–0.39)
6-month	4.31 (3.60–5.02)	4.00 (3.20–4.80)			−0.09 (−0.99- 0.80)	0.16 (−0.39–0.70)
12- month	4.29 (3.65–4.93)	3.86 (2.93–4.78)	−0.13 (0.84 -0.58)		−0.00 (−0.73-0.73)	0.21 (−0.33–0.76)

^*1*^ Testing for overall difference in level between early and late groups (main effect of group), change over time (main effect of time), and difference in time course between groups (group × time interaction). Repeated-measures ANOVA was used to account for within-subject correlation.

* number of cases may deffer from analysis to analysis.

#### SNAP management

The repeated measure analyses of variance comparing the intervention and control groups on all five management scores at baseline, six and twelve months ( Table 2) showed significant group-by-time interaction effects for nurses’ management of smoking, physical activity and weight. Post hoc analyses of the interaction effect indicated that there were differences in linear trends over time. Scores for nurses’ management of smoking, physical activity and weight increased significantly over time but those in the control group did not change. Nurses at the early intervention sites reported a significant increase in confidence related to performing SNAP interventions, especially in conducting motivational interviewing, which showed the greatest improvement.

#### Referrals to other service providers for lifestyle risk factor management

We compared the frequencies of referrals made in the intervention versus the control groups. There were no changes in the referral rate from baseline to six months and from baseline to twelve months in either intervention or control sites, except at intervention sites for referrals of overweight and obese clients for physical activity to manage their weight (see Table 2). This increased level of referrals was maintained at 12 months.

#### Changes in nurses’ confidence and perceptions of barriers and their skill in implementing lifestyle risk factor management

Nurse confidence in conducting SNAP assessment increased in the intervention sites but not in the control sites (Table 3).

There were no overall group differences in nurse’s perception of the importance of barriers over time except for a significant linear trend for a decrease in ‘*client and support related barriers’* (e.g. *lack of client interest in lifestyle changes; lack of appropriate education materials for clients*) in the intervention group. We further examined the interaction effect with post hoc analyses (see Table 4). There was a positive linear trend over time in the intervention sites and no linear trend (scores did not change) for the control group. In the intervention group the nurses’ perception of the importance of ‘client and support related barriers’ decreased from baseline to six months and this was maintained at twelve months.

### Focus group study

Of the 71 nurses at early intervention sites who attended training, 31 (43.7%) participated in a focus group. Characteristics of participating nurses are shown in Table 5. To protect the identities of individual nurses, further socio-demographic information of participants is not reported.

**Table 3:** Nurses’ confidence in assessment and management of SNAPW risk factors

		**Group mean (95% confidence interval) intervention control**		Intervention sites paired t-test*mean difference (95% CI)	Control sites paired t-test*mean difference (95% CI)	**Effect size (95% CI)**
Confidence in Assessment		(*n* = 32)	(*n* = 22)	ΔT_0_ T_1_ : ΔT_0_ T_2_	ΔT_0_ T_1_ : ΔT_0_ T_2_	
Nicotine dependence	baseline	2.44 (1.92-2.96)	2.91 (2.33-3.49)			−0.32 (−0.86–0.23)
	6-month	3.19 (2.71-3.67)	2.67 (2.07-3.27)	***0.75 (0.35 – 1.15)***	−0.14 (−0.63-0.34)	0.37 (−0.18–0.91)
	12- month	3.09 (2.63-3.55)	2.68 (2.01-3.36)	−0.09 (−0.43-0.24)	−0.05 (−0.61-0.52)	0.28 (−0.27–0.82)
Nutrition	Baseline	2.84 (2.50-3.19)	3.18 (2.64-3.72)			−0.30 (−0.84–0.25)
	6-month	3.75 (3.45-4.05)	3.05 (2.452-3.65)	***0.91 (0.58 – 1.23)***	−0.05 (−0.54-0.44)	0.62 (0.05–1.16)
	12- month	3.63 (3.35-3.90)	3.14 (2.55-3.72)	−0.13 (−0.36-0.11)	0.05 (−0.37-0.47)	0.45 (−0.10–1.00)
Weight (waist)	Baseline	3.41 (2.91-3.90)	3.73 (3.18-4.28)			−0.23 (−0.77–0.32)
	6-month	3.84 (3.45-4.23)	3.95 (3.51-4.40)	0.44 (−0.05-0.92)	0.29 (−0.26-0.83	−0.10 (−0.64–0.44)
	12- month	3.44 (2.97-3.90)	3.91 (3.44-4.38)	***−0.41 (−0.72 - -0.09)***	−0.05 (−0.41-0.32)	−0.37 (−0.92–0.18)
Risky drinking	Baseline	2.78 (2.34-3.22)	3.09 (2.51- 3.67)			−0.23 (−0.78–0.31)
	6-month	3.50 (3.12-3.88)	3.14 (2.60-3.69)	***0.72 (0.37-1.06)***	0.14 (−0.46-0.74)	0.30 (−0.25–0.85)
	12- month	3.56 (3.21-3.92)	3.32 (2.78-3.85)	0.06 (−0.20-0.32_	0.14 (−0.36-0.65)	0.21 (−0.34–0.75)
PA	Baseline	2.84 (2.45-3.23)	3.45 (3.01-3.90)			−0.55 (−1.10–0.01)
	6-month	3.59 (3.32-3.87)	3.62(3.13-4.11)	***0.77 (0.39-1.16)***	0.24 (−0.22-0.69)	−0.03 (−0.57–0.51)
	12- month	3.66 (3.40-3.91)	3.36 (2.84-3.89)	0.06 (−0.20-0.32)	−0.29 (−0.86-0.29)	0.30 (−0.25–0.85)
Readiness to change	Baseline	3.06 (2.55-3.59)	3.36 (2.88-3.85)			−0.22 (−0.76–0.33)
	6-month	3.59 (3.31-3.88)	3.00 (2.48-3.52)	0.53 (−0.03-1.10)	−0.29 (−0.85-0.27)	0.58 (0.02–1.13)
	12- month	3.55 (3.25-3.85)	3.24 (2.76-3.71)	−0.07 (−0.33-0.20)	0.15 (−0.48-0.78)	0.31 (−0.24–0.86)
Motivation Interview	Baseline	2.28 (1.78-2.78)	2.45 (1.86-3.05)			−0.12 (−0.66–0.43)
	6-month	3.16 (2.72-3.60)	2.19 (1.62-2.76)	***0.88 (0.39-1.36)***	−0.24 (−0.74-0.26)	0.74 (0.17–1.29)
	12- month	3.19 (2.82-3.57)	2.50 (1.91-3.09)	0.03 (−0.26 -0.32)	0.24 (−0.39-0.86)	0.56 (0.00–1.11)
Confidence in management						
Set goals	Baseline	2.53 (2.11-2.95)	2.45 (1.99-2.92)			0.07 (−0.48–0.61)
	6-month	3.22 (2.86-3.58)	2.33 (1.77-2.90)	***0.69 (0.24-1.13)***	−0.10 (−0.45-0.71)	0.76 (0.19–1.31)
	12- month	3.26 (2.87-3.65)	2.55 (2.00-3.09)	0.03 (−0.24-0.31)	0.14 (−0.42-0.71)	0.59 (0.03–1.14)
Smoking	Baseline	2.50 (2.02–2.98)	2.86 (2.35-3.38)			−0.27 (−0.81–0.28)
	6-month	3.28 (2.82-3.74)	2.52 (2.01-3.04)	***0.78 (0.29-1.27)***	−0.33 (−0.75-0.08)	0.59 (0.03–1.13)
	12- month	3.03 (2.60-3.47)	2.86 (2.33-3.40)	−0.26 (−0.54-0.03)	0.29 (−0.20-0.77)	0.13 (−0.41–0.68)
Nutrition	Baseline	2.81 (2.42-3.21)	3.14 (2.72-3.55)			−0.31 (−0.85–0.25)
	6-month	3.66 (3.32-3.99)	3.10 (2.54-3.65)	***0.84 (0.44-1.24)***	0.0 (−0.59-0.59	0.50 (−0.06–1.04)
	12- month	3.29 (2.96-3.62)	3.14 (2.70-3.58)	**−0.39 (−0.67- -0.11)**	0.0 (−0.41-0.41)	0.15 (−0.39–0.69)
PA	Baseline	2.66 (2.27-3.04)	3.14 (2.77-3.51)			−0.47 (−1.01–0.09)
	6-month	3.44 (3.08-3.79)	3.10 (2.62-3.57)	***0.78 (0.34-1.23)***	−0.05 (−0.63-0.54)	0.32 (−0.23–0.86)
	12- month	3.32 (3.03-3.61)	3.09 (2.62-3.56)	−0.13 (−0.41-0.15)	0.0 (−0.32-0.32	0.24 (−0.31–0.78)
Reduce Alcohol	Baseline	2.44 (1.98-2.89)	2.59 (2.17-3.02)			−0.12 (−0.67–0.42)
	6-month	3.13 (2.68-3.57)	2.30 (1.75-2.85)	***0.69 (0.19-1.18)***	−0.15 (−0.70-0.40	0.64 (0.08–1.19)
	12- month	2.90 (2.48-3.33)	2.71 (2.17-3.26)	−0.23 (−0.61-0.16)	0.21 (−0.34-0.76)	0.15 (−0.39–0.69)

* number of cases may deffer from analysis to analysis.

**Table 4:** Nurses’ perception of importance of barriers in lifestyle risk factor intervention

	**Group mean (95% confidence interval)**		***p***^**1**^			**Effect size(95% CI)**
	***Intervention***	***Control***	***Group***	***Time***	***Group × time interaction***	
**Perception of barriers**						
**Client- & support related**	(*n* = 32)	(*n* = 21)	0.368	**0.050**	**0.010**	
Baseline	3.05 (2.82-3.28)	2.85 (2.51 -3.19)				0.28 (−0.28-0.83)
6-month	2.51 (2.23-2.78)	2.97 (2.73 - 3.22)				−0.65 (−1.20--0.07)
12-month	2.63 (2.37-2.89)	2.77 (2.45 - 3.08)				−0.19 (−0.74-0.37)
**Clinician-related**	(*n* = 32)	(*n* = 20)	0.663	0.543	0.248	
Baseline	2.19 (1.85-2.53)	1.85 (1.43 - 2.27)				0.35 (−0.22-0.91)
6-month	2.00 (1.64-2.36)	2.13 (1.69 - 2.57)				−0.13 (−0.68-0.43)
12-month	1.94 (1.63-2.25)	1.88 (1.49 - 2.27)				0.07 (−0.49-0.63)
**Service-related**	(*n* = 30)	(*n* = 21)	0.417	0.853	0.411	
Baseline	2.87 (2.49-3.24)	2.97 (2.63 - 3.31)				−0.10 (−0.66-0.45)
6-month	2.77 (2.54-3.01)	3.10 (2.79 - 3.40)				−0.48 (−1.04-0.09)
12-month	2.97 (2.80-3.14)	2.87 (2.52 - 3.22)				0.16 (−0.40-0.71)

^*1*^ Testing for overall difference in level between early and late groups (main effect of group), change over time (main effect of time), and difference in time course between groups (group × time interaction). Repeated-measures ANOVA was used to account for within-subject correlation.

**Table 5:** Make-up of focus groups

**Focus Group**	**Age group (years)**	**Gender**	**No. of participants**	**Average years working as community nurses (range)**
Focus group 1	18 – 55	Female only	9	7.7 (0.5 – 21)
Focus group 2	35 – 55	Female only	3^a^	3.0 (0.5 – 7)
Focus group 3	18 – 65	Mixed^b^	7	5.9 (0.5 -20)
Focus group 4	18 – 65	Female only	12	9.8 (1–20)

^a^This was a small group but all three participants contributed actively to the discussion.

^b^There were 2 male nurses and 5 female nurses in this group.

#### Assessment

Most nurses, especially new or less experienced ones, considered the formalised approach to SNAP assessment to be useful. It was acknowledged that the intervention was “an extension” to the work that these nurses routinely perform.

“We’ve actually had a similar type of informal questions along the same line anyway so we were already using a similar system … SNAP’s has been a more formal presentation and probably would help a lot of less experienced nurses have a gauge on where to go with information to give people.” (Nurse 2, FG^*a*^4)

This view was echoed by one of the nurse managers:

“It was really just an extension of what they were already doing and in many ways just formalised something that was part of our everyday practice.” (NUM^*b*^1)

#### Brief interventions

There were various opinions as to the feasibility of delivering brief lifestyle interventions. These reflected differences in the way nurses perceived their roles and the way they implemented lifestyle interventions. Some nurses preferred addressing lifestyle issues over a few consultations as there was a danger of overloading clients with health information if it was all presented at the first meeting. Also, it was argued that a second or subsequent consultation allowed clients to be better engaged with preventive health issues.

“If we discussed maybe the one aspect of it [intervention]maybe at each visit or every second visit that people were more inclined to listen and maybe want to change, but trying to do it all in the first visit…, that’s too hard that – no, they just want me to do their dressing.” (Nurse 2, FG2)

Despite generally seeing brief lifestyle interventions as fitting well with their role, a number of nurses believed that there was also a limit to the extent to which they could provide lifestyle interventions. This was primarily related to time pressures and the need to address the immediate health concern that was the reason for the nurse’s visit.

“I think we have a duty of care. If no-one has approached these topics with the person in previous times then I think we should definitely go over it with them… maybe, yeah, have a bit of a chat about it ‘Hey how’d you go with that walking group?’ when we go to their home visit.” (Nurse 3, FG3)

Time constraints and a large caseload were identified by both nurses and managers as a major barrier to conducting brief lifestyle interventions:

“I think that a lot of the pressures on the nurses are around time… they would say that it’s difficult to fit that into their daily practice and I think you need to see some degree of commitment and personal commitment… for them [nurses] to be able to achieve it.” (NUM 1)

“We’ve got a very big caseload… and you can’t always sit down and have a nice little chat about what they’re eating because you’re just too busy so we’re not really given enough time to do that all the time.” (Nurse 6, FG1)

#### Referral

Feedback from the nurses suggests that they were aware of the need to refer clients to support groups for longer-term behaviour change maintenance but a lack of referral services and long waiting lists were cited as barriers:

“When you ask them would they be interested in more exercise and you might be able to guide them in different ways on how they can start building their exercise up. But it would be really handy if we had somewhere that we could have a referral where we could refer them to and where they did have these exercise groups.” (Nurse 5, FG3)

“Public system physiotherapy is another significant waiting list; they can get private… but the hospital physio is a difficult one.” (Nurse 5, FG4)

However, for referrals to be taken up by clients, nurses also suggested rotating these support group initiatives throughout the different areas and providing free transport for clients to attend such SNAP programs:

“Provide transport, have it local for so many weeks and then move it to another area and have it there for so many weeks so that there’s more access to it and everyone’s being provided with similar education. That would be the motivation to go onto the next step of whatever might need to happen.” (Nurse 6, FG4)

#### Changes in nurses’ perceptions of barriers and their skill in implementing lifestyle risk factor management

The perceived importance of ‘*nurse-related barriers’* (e.g. *personal lack of interest in addressing lifestyle risk factors; my own lifestyle habits*) and *‘system-related barriers’* (e.g. *pressures of providing post-acute care; short-term contact with clients*), remained the same across time.

Nurses saw themselves as having the skills to talk to clients and provide guidance about lifestyle issues.

“The motivational thing [interview] and if we, any of us, run any clinic we do that ourselves anyway, so I think most of us feel very comfortable being able to speak to our clients about any aspect really.” (Nurse 2, FG2)

## Discussion

We found that a team-based intervention that focused on building positive attitudes, skills and self-efficacy of nurses, as well as providing organisational level support was effective in improving self-reported lifestyle risk factor assessment and management practices of community nurses but had little impact on rates of referral.

It is likely that the use of standardised assessment forms and nurses’ increased confidence in these aspects of lifestyle risk factor management helped improve the frequency of assessment for physical activity, nutrition and weight. Assessment for smoking and alcohol consumption showed a ceiling effect in both intervention and control sites, probably due to the pre-existing high level of awareness of the need to assess for smoking and at-risk drinking behaviour.

The increased frequency at which nurses provided brief interventions for physical activity, nutrition and weight may reflect the increased identification of these risk factors through more frequent assessments. Increases in the provision of brief interventions for all lifestyle risk factors (with the exception of alcohol) may also be explained by a boost in clinicians’ confidence and a reduction in their perception of ‘client and support related’ barriers as the trial progressed. The reduction in client and support related barriers may reflect the nurse training as well as the decision support tools and resources provided (including the integration of standardised assessment tools into routine assessment processes, written actions plans for clients and a written guide and referral directory).

Despite the increased awareness of the importance of referral by nurses, rates of actual referrals to other service providers for more intensive or longer-term management remained low in both the intervention and control groups, except for the increased rates of referral to physical activity programs for obese clients in the intervention group at six and twelve months. It was apparent that nurses encountered significant barriers to referring, including a lack of local services to refer to, poor access to group programs, transport difficulties and long waiting lists for services. This finding suggests the need to develop more effective linkages with other services and collaborative strategies to remove access barriers.

### Limitations of the trial

One of the unavoidable limitations of the study was that recorded nurse practice was based on self-report, which might encourage socially desirable responses. Moreover, nurses from control sites commented that simply answering the survey prompted them to be more aware of the need to include addressing lifestyle risk factors in their professional care of their clients (i.e. the Hawthorn effect) [28]. This study adopted a quasi-experimental design because it was not feasible to randomise the intervention according to individual patients or practitioners within the services. The overall response rate of the survey across all time points was only 41.9% (54/129), which would also affect the generalisability of the findings, as nurses interested in lifestyle risk factor management might have been more likely to participate in the study.

## Conclusion

In this study a comprehensive team-based intervention was associated with improvements in the lifestyle risk factor management practices of GCHNs, particularly in relation to the use of assessment and brief interventions to support improved physical activity, decreased smoking and improved nutrition/weight. The sustainability needs to be explored; unless lifestyle risk factor management is incorporated into new service models it is likely to be eroded by other work pressures. The effectiveness of the intervention likely reflects the improved training and supports provided to nurses, but time pressures resulting from the prevailing model of care, with its focus on management of acute problems, present an important potential threat to maintaining such practices over time. Significant barriers remain to improving the rates of referral for lifestyle risk factors. This area requires further attention given the importance of referral to more intensive interventions for achieving long-term changes in behaviour and reductions in chronic disease.

## End note

^a^FG – focus group.

^b^NUM – nursing unit manager.

## Competing interests

The authors declare that they have no competing interests.

## Authors’ contributions

BC contributed to design of survey, data collection and analysis, revision of the focus group schedule, qualitative data collection, data analysis, data interpretation, and drafting the manuscript. UJ contributed to the conception of study, contributed to data analysis including, repeated measures analysis, writing part of the ‘Findings’, and feedback to the final draft. RL conception of study, design of survey, data analysis, data interpretation, contribute to writing ‘Introduction’ and ‘Discussion’ and feedback to the final draft. NO provided input into survey design, contributed to writing the conclusion and feedback on the final draft. AW contributed to the conception of study, design of survey, input into qualitative schedule, and contribution to the content of the paper and feedback on the final draft. KP provided input into survey design, assisting with recruiting clinicians for pilot and study, feedback to earlier ideas on other paper related to clinician practice, extracting quotes from qualitative data, writing ‘Discussion’ and feedback on the final draft. MH contributed to the conception of study, design of survey, overseeing and contributing to the writing of this paper. All authors read and approved the final manuscript.

## Pre-publication history

The pre-publication history for this paper can be accessed here:

http://www.biomedcentral.com/1472-6963/13/54/prepub

## Supplementary Material

Additional file 1 Community nursing SNAP trial- survey of assessment and management for lifestyle risk factors. Click here for file
