# Supplementary material for: The impact of a team-based intervention on the lifestyle risk factor management practices of community nurses: outcomes of the community nursing SNAP trial
Source: BMC Health Serv Res. 2013 Feb 9;13:54. doi: 10.1186/1472-6963-13-54 (PMC3599701; doi:10.1186/1472-6963-13-54)
Supplement: Additional file 1 — Community nursing SNAP trial- survey of assessment and management for lifestyle risk factors. [file 1472-6963-13-54-S1.doc]

**Community Nursing SNAP Trial- Survey of Assessment and Management for Lifestyle Risk Factors**

This survey takes approximately 20 minutes to complete. All information that you provide will be kept **confidential** and stored securely. This survey is for research purpose only, it will be de-identified, your name will not be known and your individual results will not be reported. It will **NOT** be used as an appraisal of your clinical performance.

**1. Clinician details**

- 1. **Which of the following area health services[[1]](#footnote-2) do you work for?**

❑ A-AHS ❑ B-AHS ❑ C-AHS

- 1. **Gender:** ❑ Female ❑ Male

**1.3 Age:** ❑ 18-24 ❑ 25-34 ❑ 35-44 ❑ 45-54 ❑ 55-64 ❑ 65+

**1.4 Working status**: ❑ Full-time ❑ Part-time ❑ Casual

If part-time, how many hours do you work per week? _______

- 1. **Clinician type:** ❑ Registered Nurse ❑ Enrolled Nurse

- 1. **How long** have you worked in **community nursing**? ____________

- 1. How long have you worked in **this** team/service? _______________

# How often in the **last 2 months** did you **ask new clients about** the following risk factors:

|  | **Never** | **Rarely** | **Sometimes** | **Half the time** | **Often** | **Usually** | **Always** |
| --- | --- | --- | --- | --- | --- | --- | --- |
| % of clients | *0%* | *1-20%* | *21-40%* | *41-60%* | *61-80%* | *81-99%* | *100%* |
| Diet |  |  |  |  |  |  |  |
| Weight |  |  |  |  |  |  |  |
| Physical Activity |  |  |  |  |  |  |  |
| Smoking status |  |  |  |  |  |  |  |
| Alcohol consumption |  |  |  |  |  |  |  |
| Other comments: | | | | | | | |

1. How often on average do you do the following when dealing with a client with a **poor diet?**

|  | **Never** | **Rarely** | **Sometimes** | **Half the time** | **Often** | **Usually** | **Always** |
| --- | --- | --- | --- | --- | --- | --- | --- |
|  | *0%* | *1-20%* | *21-40%* | *41-60%* | *61-80%* | *81-99%* | *100%* |
| Ask the client about their interest in making dietary changes |  |  |  |  |  |  |  |
| Advise about dietary recommendations |  |  |  |  |  |  |  |
| Discuss strategies to assist the client make dietary changes |  |  |  |  |  |  |  |
| Provide written information |  |  |  |  |  |  |  |
| Refer to a dietitian or other support service or program |  |  |  |  |  |  |  |
| Follow up progress in subsequent visits |  |  |  |  |  |  |  |
| Other comments: | | | | | | | |

# How often on average do you do the following when dealing with a **client who smokes**?

#

|  | **Never** | **Rarely** | **Sometimes** | **Half the time** | **Often** | **Usually** | **Always** |
| --- | --- | --- | --- | --- | --- | --- | --- |
|  | *0%* | *1-20%* | *21-40%* | *41-60%* | *61-80%* | *81-99%* | *100%* |
| Advise the client to quit smoking |  |  |  |  |  |  |  |
| Ask the client about their interest in quitting |  |  |  |  |  |  |  |
| Assess nicotine dependency |  |  |  |  |  |  |  |
| Set a quit date |  |  |  |  |  |  |  |
| Recommend nicotine replacement therapy |  |  |  |  |  |  |  |
| Refer to Quitline or other smoking cessation program |  |  |  |  |  |  |  |
| Provide written information |  |  |  |  |  |  |  |
| Discuss other strategies to assist the client to quit |  |  |  |  |  |  |  |
| Follow up progress at subsequent visits |  |  |  |  |  |  |  |
| Other comments: | | | | | | | |

# How often on average do you do the following when dealing with an **overweight or obese client?**

|  | **Never** | **Rarely** | **Sometimes** | **Half the time** | **Often** | **Usually** | **Always** |
| --- | --- | --- | --- | --- | --- | --- | --- |
|  | *0%* | *1-20%* | *21-40%* | *41-60%* | *61-80%* | *81-99%* | *100%* |
| Ask the client about their interest in losing weight |  |  |  |  |  |  |  |
| Advise of the benefits of a 5-10% weight loss |  |  |  |  |  |  |  |
| Set a goal for weight loss |  |  |  |  |  |  |  |
| Advise the client to eat less dietary fat |  |  |  |  |  |  |  |
| Advise the client to eat more fruit and vegetables |  |  |  |  |  |  |  |
| Advise the client to do more physical activity |  |  |  |  |  |  |  |
| Discuss strategies to assist the client to improve their diet and do more physical activity |  |  |  |  |  |  |  |
| Provide written information |  |  |  |  |  |  |  |
| Refer to a dietitian or other support service or program |  |  |  |  |  |  |  |
| Refer to a physical activity program |  |  |  |  |  |  |  |
| Follow up progress in subsequent visits |  |  |  |  |  |  |  |
| Other comments: | | | | | | | |

# How often on average do you do the following when dealing with a **client with ‘at-risk’ alcohol consumption (drinking more than recommended)?**

#

|  | **Never** | **Rarely** | **Sometimes** | **Half the time** | **Often** | **Usually** | | **Always** |
| --- | --- | --- | --- | --- | --- | --- | --- | --- |
|  | *0%* | *1-20%* | *21-40%* | *41-60%* | *61-80%* | *81-99%* | | *100%* |
| Ask the client about their interest in reducing their alcohol intake |  |  |  |  |  |  |  | |
| Advise the client about the recommended alcohol intake |  |  |  |  |  |  |  | |
| Advise the client to reduce alcohol intake |  |  |  |  |  |  |  | |
| Discuss strategies to assist the client to reduce alcohol intake |  |  |  |  |  |  |  | |
| Refer to drug and alcohol counsellor or other support service or program |  |  |  |  |  |  |  | |
| Provide written information |  |  |  |  |  |  |  | |
| Follow up progress at subsequent visits |  |  |  |  |  |  |  | |
| Other comments: | | | | | | | | |

# How often on average do you do the following when dealing with a client with **inadequate levels of physical activity** (excluding those with physical limitations)?”

#

|  | **Never** | **Rarely** | **Sometimes** | **Half the time** | **Often** | **Usually** | **Always** |
| --- | --- | --- | --- | --- | --- | --- | --- |
|  | *0%* | *1-20%* | *21-40%* | *41-60%* | *61-80%* | *81-99%* | *100%* |
| Ask the client about theirinterest in doing more physicalactivity |  |  |  |  |  |  |  |
| Advise the client about therecommended level of physical activity |  |  |  |  |  |  |  |
| Advise the client to do more physicalactivity |  |  |  |  |  |  |  |
| Discuss strategies to assist the clientto do more physical activity |  |  |  |  |  |  |  |
| Provide written information |  |  |  |  |  |  |  |
| Refer to a exercise physiologist,  physiotherapist or physical activity  program |  |  |  |  |  |  |  |
| Follow up progress at subsequent visits |  |  |  |  |  |  |  |
| Other comments: | | | | | | | |

1. **When you needed to, how often were you able to find accessible services, providers or support groups to refer to for the lifestyle risk factors listed?**

|  | **Never** | **Rarely** | **Sometimes** | **Half the time** | **Often** | **Usually** | **Always** |
| --- | --- | --- | --- | --- | --- | --- | --- |
|  | *0%* | *1-20%* | *21-40%* | *41-60%* | *61-80%* | *81-99%* | *100%* |
| Poor Diet /Nutrition |  |  |  |  |  |  |  |
| Overweight/obesity |  |  |  |  |  |  |  |
| Physical inactivity |  |  |  |  |  |  |  |
| Smoking |  |  |  |  |  |  |  |
| ‘At risk’ Alcohol consumption |  |  |  |  |  |  |  |
| Other comments: | | | | | | | |

1. When you provide advice about lifestyle risk factors, **how much time** do you estimate that you spend on average addressing each of the following:

|  | **Time in minutes** | **Do not provide advice** |
| --- | --- | --- |
| Diet /Nutrition |  |  |
| Physical Activity |  |  |
| Smoking |  |  |
| Alcohol |  |  |
| Other comments: |  |  |

1. Clients I see find it **acceptable** for me **to raise** the following lifestyle issues routinely as part of the consultation:

|  | **Strongly**Agree | **Somewhat**Agree | **Neither** Agree **Nor** Disagree | **Somewhat** Disagree | **Strongly** Disagree | **Do not discuss** |
| --- | --- | --- | --- | --- | --- | --- |
| Smoking |  |  |  |  |  |  |
| Alcohol |  |  |  |  |  |  |
| Nutrition |  |  |  |  |  |  |
| Weight |  |  |  |  |  |  |
| Physical activity |  |  |  |  |  |  |
| Other comments: | | | | | | |

# Please rate your **confidence** in undertaking the following activities with clients:

|  | **Not at all**Confident | **Minimally**Confident | **Somewhat**Confident | **Moderately**Confident | **Very**Confident |
| --- | --- | --- | --- | --- | --- |
| Assessing nicotine dependence |  |  |  |  |  |
| Assessing nutrition |  |  |  |  |  |
| Measuring waist circumference |  |  |  |  |  |
| Assessing risky drinking |  |  |  |  |  |
| Assessing physical activity |  |  |  |  |  |
| Assessing readiness to change |  |  |  |  |  |
| Undertaking motivational interviewing |  |  |  |  |  |
| Setting goals for lifestyle change |  |  |  |  |  |
| Helping clients quit smoking |  |  |  |  |  |
| Helping clients improve their eating habits |  |  |  |  |  |
| Helping clients become more physically active |  |  |  |  |  |
| Helping clients reduce alcohol consumption |  |  |  |  |  |
| Other comments: | | | | | |

# How **important** are the following potential **barriers to addressing lifestyle risk factors** in your work?

|  | **Very**Important | **Moderately**Important | **Somewhat**Important | **Not very**Important |
| --- | --- | --- | --- | --- |
| Lack of time |  |  |  |  |
| Pressures of providing post acute care |  |  |  |  |
| Lack of relevance to the clients’ presenting issue |  |  |  |  |
| Lack of relevance to my role |  |  |  |  |
| Personal lack of interest in addressing lifestyle risk factors |  |  |  |  |
| Lack of client interest in lifestyle changes |  |  |  |  |
| Lack of opportunity to talk about lifestyle risk factors with clients |  |  |  |  |
| Lack of appropriate education materials for clients |  |  |  |  |
| Short term contact with clients |  |  |  |  |
| Limited availability or access to support services to help clients with lifestyle change |  |  |  |  |
| Communication difficulties with clients |  |  |  |  |
| Cultural differences between nurses and clients |  |  |  |  |
| My own lifestyle habits |  |  |  |  |
| Other comments: | | | | |

# To what extent do you **agree** with the following statements?

|  | **Strongly**Agree | **Somewhat**Agree | **Neither** Agree **Nor** Disagree | **Somewhat** Disagree | **Strongly** Disagree |
| --- | --- | --- | --- | --- | --- |
| Helping clients make lifestyle changes is an essential part of my role as a community nurse |  |  |  |  |  |
| Lifestyle issues are best addressed by qualified experts or specialist services |  |  |  |  |  |
| Lifestyle issues are best tackled at a community or population level rather than through individual intervention |  |  |  |  |  |
| I have access to tools to help me decide if and how to provide lifestyle intervention to clients |  |  |  |  |  |
| Health promotion activities are valued by management as an important part of the community nurses role |  |  |  |  |  |
| Clients I see are generally not motivated to make lifestyle changes |  |  |  |  |  |
| Clients I see are generally too old to benefit from lifestyle change |  |  |  |  |  |
| For most clients, health education does little to promote their adherence to a healthy lifestyle |  |  |  |  |  |
| Clients without symptoms will rarely change their behaviour on the basis of my advice |  |  |  |  |  |
| Most clients try to change their lifestyles if I advise them to do so. |  |  |  |  |  |
| Providing lifestyle advice is an effective use of my time as a clinician |  |  |  |  |  |
| Other comments: | | | | | |

# In the past 2 years have you had training on any of the following?

|  | **Yes (please specify­)** | **No** |
| --- | --- | --- |
| Smoking |  |  |
| Diet / Nutrition |  |  |
| Alcohol |  |  |
| Physical activity |  |  |
| Motivational interviewing |  |  |
| Assessing clients’ readiness to change |  |  |
| Client education strategies |  |  |
| Other comments: | | |

**14.1** Would you like more training in assisting clients to manage lifestyle risk factors?

❑ Yes please describe: _________________________________ ❑ No

# **14.2** If you would like training, in what format would you like to receive it? (tick all that apply)

❑ Workshop ❑ clinical supervision / mentoring ❑ self-study materials

❑ Case studies ❑ small group discussions ❑ Other: _____________

# Any other comments:___________________________________________________________

**______________________________________________________________________________________________________________**

**________________________________________________________________________________________**

1. *Names of Area Health Services were not provided here to protect confidentiality of participants* [↑](#footnote-ref-2)
